# NFATc1 Abrogation in B Cells Ameliorates Contact Hypersensitivity Responses

**DOI:** 10.3390/ijms26178125

**Published:** 2025-08-22

**Authors:** Franziska Grän, Muhammad Azeem, Edgar Serfling, Matthias Goebeler, Andreas Kerstan, Khalid Muhammad

**Affiliations:** 1 Department of Dermatology, Venereology and Allergology, University Hospital Würzburg, 97080 Würzburg, Germany; franziska.graen@uk-augsburg.de (F.G.); azeem_m@ukw.de (M.A.); goebeler_m1@ukw.de (M.G.); 2 Department of Molecular Pathology, Institute of Pathology, University of Würzburg, 97078 Würzburg, Germany; serfling.e@uni-wuerzburg.de; 3 Department of Biology, College of Science, United Arab Emirates University, Al Ain 15551, United Arab Emirates

**Keywords:** B-lymphocytes, contact hypersensitivity, interleukin-10, NFATc1 transcription factor, regulatory B-lymphocytes

## Abstract

Allergic contact dermatitis (ACD) is a frequent inflammatory skin disease that evolves upon exposure to contact allergens in sensitized individuals. Both the adaptive and innate immune system play pivotal roles in the pathogenesis of ACD. While the importance of T cells is undisputed, the relevance of B lymphocytes is less clear. The published data support a critical role for NFATc1 in B cell activation. Therefore, we investigated the impact of NFATc1 on B cell function during murine contact hypersensitivity (CHS), the mouse model for human ACD. Compared with wild-type mice, B cell-specific ablation of NFATc1 (*Nfatc1^f/f^ x mb1-cre*) resulted in significantly diminished CHS responses measured by ear thickness (0.81 ± 0.02 mm vs. 0.48 ± 0.02 mm (*p* = 0.0007)) to the obligate contact allergen 2,4,6-trinitrochlorobenzene, accompanied by a marked increase in the frequency of IL-10-producing regulatory B cells. Flow cytometric analysis showed that IL-4- and IL-17-producing CD4^+^ T cells were reduced, while IFN-γ-producing CD4^+^ T cells were marginally increased in *Nfatc1^f/f^ x mb1-cre* mice. In conclusion, NFATc1 mediates CHS responses by modulating the development of IL-10-producing B cells. These findings support the compelling notion that targeting NFATc1 may represent a potential therapeutic strategy for allergic responses.

## 1. Introduction

Allergic Contact Dermatitis (ACD) is a common inflammatory skin disorder resulting from a delayed-type (Type IV) hypersensitivity reaction that develops upon skin exposure to allergens [1]. Erythema, edema, vesiculation, and pruritus evolve after 48–72 h at the contact site and chronic progression of the disease can occur when patients are exposed repeatedly or continuously to the allergens [2]. Metals, especially nickel, cobalt and dichromate, preservatives such as formaldehyde, acrylates and para-phenylenediamine, but also fragrances may act as allergens causing ACD [3]. Sources of these allergens are often substances of daily usage such as consumer products, leather, hair dyes and cosmetics, making it sometimes difficult to avoid them in daily life and at the workplace [4]. In an occupational setting, the elimination of these agents can be problematic and, in some cases, impossible. Consequently, ACD has a major impact on the quality of life in affected patients [5].

The pathophysiology of ACD has been extensively studied. In contrast to irritant contact dermatitis (ICD), ACD is characterized by a sensitization phase followed by activation of the adaptive immune system upon re-exposure to the allergen. The allergens responsible for this reaction are low-molecular-weight chemicals, known as haptens, and require epidermal proteins as an adjuvant to induce allergic reactions [6,7,8]. For this reason, most haptens are lipophilic so that they can penetrate the epidermis easily. During the initial sensitization phase, cutaneous dendritic cells (DCs) process the protein-conjugated haptens and migrate to the regional lymph nodes where they act as antigen-presenting cells (APCs). They sensitize and activate CD8^+^ and CD4^+^ cells, generating antigen-specific T cells [9]. Of interest, innate immune responses directly elicited by allergens orchestrate the sensitization and elicitation phase, as has been shown for ACD to nickel [7,10,11,12]. Upon second exposure to the same allergen, the elicitation phase with clinically apparent symptoms and skin lesions evolves. During this process, APCs present a hapten–protein complex on MHC molecules, which activates antigen-specific lymphocytes in the dermis, epidermis and draining lymph nodes. This results in the recruitment of even more immune cells culminating in inflammatory processes, which are ultimately responsible for clinical symptoms [6,13].

Nuclear Factor of Activated T Cell (NFAT) transcription factors are pivotal switch factors in the human immune system. Similar to its expression in peripheral T cells [14,15], due to the use of two alternate promoters and a poly A site and alternate splicing events, the *Nfatc1* gene is expressed in six prominent isoforms in peripheral B cells [15]. It has been previously shown that NFATc1 plays a role in B cell development and protection against Imiquimod-induced psoriasis-like skin inflammation via IL-10 [16]. Albeit a plethora of scientific data support a major role of T lymphocytes in driving the cutaneous inflammation [17] and contact hypersensitivity (CHS) is considered to be mainly driven by hapten-specific T effector cells, the importance of B-lymphocytes, especially regulatory B cells (Bregs), in controlling the allergic response is poorly understood. Watanabe et al. reported that B cells are important for the mechanisms behind ACD since CD19 loss leads to an increase and prolongation of CHS in mice, suggesting a possible downmodulation of inflammation by B cells, especially IL-10-producing Bregs [18]. Of note, it has been shown that NFATc1 might dampen the regulatory function of Bregs via the inhibition of IL-10 production [16]. Using transgenic mice deficient in NFATc1 in B cells, we aimed to explore the role of the NFATc1 transcription factor and B cells during the CHS responses.

## 2. Results

### 2.1. Contact Hypersensitivity Induction by Trinitrochlorobenzene (TNCB)

**Figure 1:**
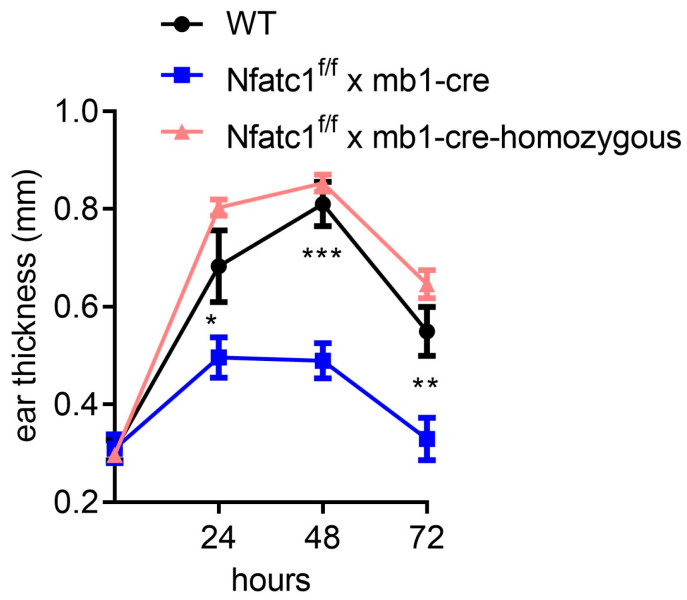
TNCB-induced Contact Hypersensitivity Response. Ear swelling was measured before and at 24, 48 and 72 h after CHS elicitation. CHS responses peaked at 48 h, with wild-type (WT) mice exhibiting significantly greater swelling compared to *Nfatc1^f/f^ x mb1-cre* mice. *** *p* < 0.001, ** *p* < 0.01, and * *p* < 0.05.

To elucidate the role of B cells in CHS in general, B cell-deficient mice (*Nfatc1^f/f^ x mb1-cre* (homozygous for mb1-cre) mice) were used. B cell-deficient mice were homozygous for mb1-cre, which bear two mutated *Mb1*/*Cd79a* alleles (encoding the B cell chain), resulting in a drastic B cell signaling defect [19]. These mice were sensitized by applying TNCB to the shaved abdominal skin and elicited five days later by application of the same hapten to the ear pinna. Subsequently, ear swelling was measured daily over a period of 3 days, revealing an increased inflammatory response in B cell-deficient animals that peaked at 48 h and persisted throughout the duration of the experiment. These data indicate that CHS responses are enhanced in B cell-deficient mice (Figure 1).

### 2.2. Mice with NFATc1 Deficiency in B Cells Exhibit Reduced CHS Responses

Next, we asked whether NFATc1 deficiency in B cells could show a differential CHS response. Interestingly, mice deficient for NFATc1 in B cells (*Nfatc1^f/f^ x mb1-cre*) presented with a significantly diminished ear swelling compared to WT mice upon TNCB elicitation (Figure 1). In detail, the ear thickness was significantly higher in WT mice compared to *Nfatc1^f/f^ x mb1-cre* mice at 24 h (0.69 ± 0.04 mm vs. 0.50 ± 0.02 mm (*p* = 0.0188)), which peaked at 48 h (0.81 ± 0.02 mm vs. 0.48 ± 0.02 mm (*p* = 0.0007)) and remained elevated at 72 h (0.55 ± 0.02 mm vs. 0.33 ± 0.02 mm (*p* = 0.004)).

**Figure 2:**
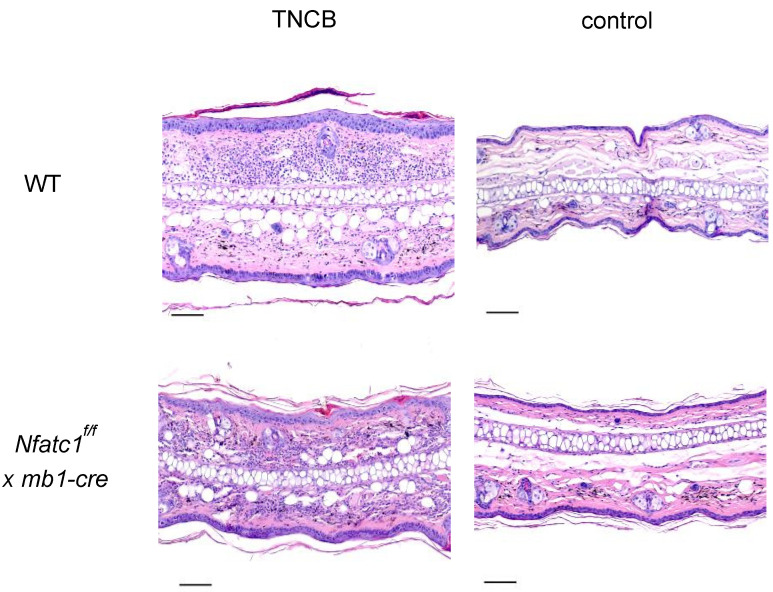
Histologic examination of CHS responses after elicitation. Ear tissue sections of wild-type (WT) and *Nfat1^f/f^ x mb1-cre* mice were collected 3 days after elicitation and stained with hematoxylin and eosin (H&E). WT mice displayed an increased dermal cell infiltrate as compared to *Nfat1^f/f^ x mb1-cre* mice during CHS responses. Non-sensitized mice which were elicitated with TNCB were used as control. Scale bar = 50 µm.

At the end of the elicitation phase, microscopic examination of ear sections obtained from *Nfatc1^f/f^ x mb1-cre* mice stained by hematoxylin and eosin (H&E) showed considerably less cellular infiltration in the dermis upon elicitation than WT animals (Figure 2).

### 2.3. IL-10-Producing Regulatory B Cells Are Enhanced in Nfat1^f/f^ x mb1-Cre Mice

**Figure 3:**
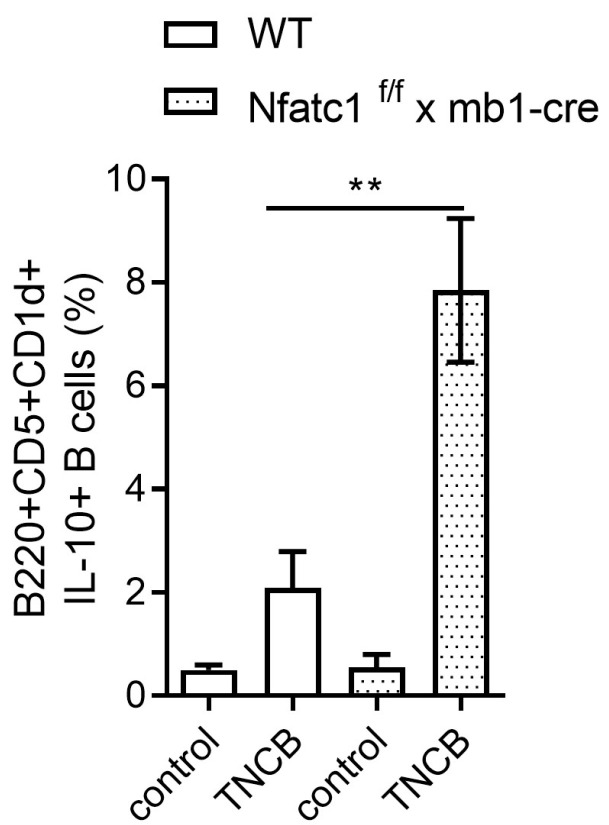
Frequency of IL-10-producing B_regs_ during CHS. After sensitization and elicitation of mice, splenic lymphocytes were isolated at day 8 and stained for IL-10^+^ Bregs. *Nfatc1^f/f^ x mb1-cre* mice showed a significantly higher frequency of IL-10^+^ Bregs during CHS responses compared to WT mice. Under control conditions, no significant differences were observed. ** *p*< 0.01.

**Figure 4:**
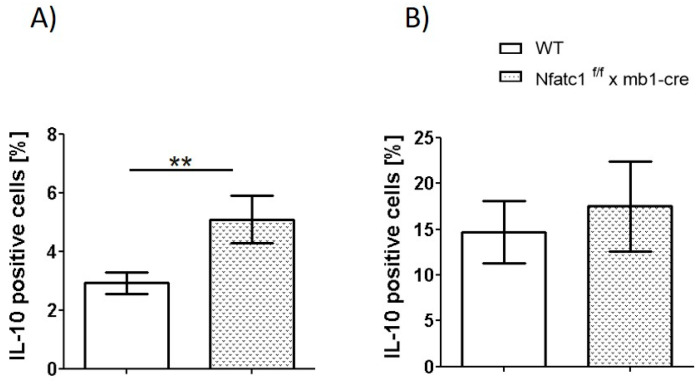
IL-10-positive B-lymphocytes in spleen (**A**) and ears (**B**) after elicitation of CHS. TNCB-sensitized mice were subjected to elicitation and at day 8, cells were isolated from the spleen and ear. In the spleen, *Nfatc1^f/f^ x mb1-cre* mice exhibited a significantly higher number of IL-10-producing B cells compared to wild-type (WT) mice. In the mice ears, IL-10^+^ B cells (B220^+^IL-10^+^) were also slightly increased, although this difference was not statistically significant. ** *p*< 0.01.

To better understand the role of B cells in CHS, we analyzed different B cell subsets in WT and *Nfatc1^f/f^ x mb1-cre* mice. The total number of splenic B cells appeared to be equal in both mice strains. The CD5^+^CD1d^high^ B cell subset has been previously characterized as a hallmark population of regulatory B cells (Bregs) known to secrete IL-10 [20]. Consistent with this, our analysis revealed a significantly elevated IL-10 production by this cell subset in *Nfatc1^f/f^ x mb1-cre* mice compared with WT mice (Figure 3). Moreover, IL-10-producing B cells (B220^+^IL10^+^ cells) were significantly increased in the spleen and non-significantly elevated Breg cells were observed in mice ears (Figure 4). Accordingly, our data of increased Breg numbers, along with increased IL-10 production, indicate an enhanced regulatory B cell function in the absence of functional NFATc1 within the B cell compartment.

### 2.4. Influence of NFATc1 Deficiency in B Cells on Cytokine Production by T Cells

To assess the impact of NFATc1-deficient B cells on T cell cytokine production during CHS responses, we analyzed IL-4–, IL-17–, and IFN-γ–producing CD4^+^ T cell subsets in the ear and spleen at day 8 of CHS. In the ear, notable differences were observed in the frequencies of IL-4^+^ and IL-17^+^ CD4^+^ T cells. Specifically, *Nfatc1^f/f^ x mb1-cre* mice showed a significantly lower percentage of IL-4^+^ CD4^+^ T cells as compared with WT controls (* *p* < 0.05). The same trend was observed for IL-17^+^ cells. However, there was no major difference regarding the number of IFN-γ^+^ cells. In the spleen, there was no major difference in the percentage of IL-4^+^ and IFN-γ^+^ cells while the percentage of IL-17^+^ cells was much lower in the *Nfatc1^f/f^ x mb1-cre* mice compared with WT mice (* *p* < 0.05).

In conclusion, these findings suggest that there are lower percentages of IL-4- and IL-17-producing CD4^+^ T cells in the ears of *Nfatc1^f/f^ x mb1-cre* mice. In the spleen, we only observed a major reduction in IL-17-producing CD4^+^ T cells in the *Nfatc1^f/f^ x mb1-cre* mice, indicating that the diminished CHS responses were also due to subtle changes in CD4^+^ T cell cytokines.

## 3. Discussion

ACD is a classical T cell-dependent delayed hypersensitivity reaction. A substantial amount of data derived from murine CHS, the animal model for human ACD, points to the contribution of different roles of CD4^+^ and CD8^+^ T cells during such allergic responses [21,22,23]. However, Bregs have also drawn attention to their contribution to various inflammatory responses through control and tolerance induction [20,24]. Immune tolerance, a fundamental mechanism that prevents excessive or misdirected immune responses, is often impaired in allergic conditions. Previous studies demonstrated that transfer of IL-10-producing B cells, but not IL-10-deficient B cells, suppressed allergic responses in mice [20,25,26]. Our study suggests that Bregs exert a suppressive role in CHS responses through mechanisms dependent on NFATc1 signaling. NFATc1 is traditionally associated with T cell activation but is increasingly recognized as a key modulator of B cell functions, particularly in regulating IL-10 production, a hallmark of Bregs [15]. Previous studies have shown that NFATc1 suppresses IL-10 expression by directly binding to its promoter and recruiting chromatin-modifying enzymes [16]. A recent study further demonstrated that NFATc1-deficient B cells ameliorate atopic dermatitis-associated allergic responses by significantly increasing IL-10-producing regulatory B cells. Direct functional evidence for the role of IL-10 was provided by adoptive transfer experiments, in which IL-10-producing B cells reduced atopic dermatitis-associated ear swelling. This study also confirmed that NFATc1 deficiency impacts MAPK pathways controlling IL-10 production, e.g., p38MAPK inhibition resulted in reduced Il10 gene transcription. Together, these findings strengthen the claim that NFATc1 negatively regulates IL-10 expression and establish IL-10’s necessity in the observed protective phenotype [27].

**Figure 5:**
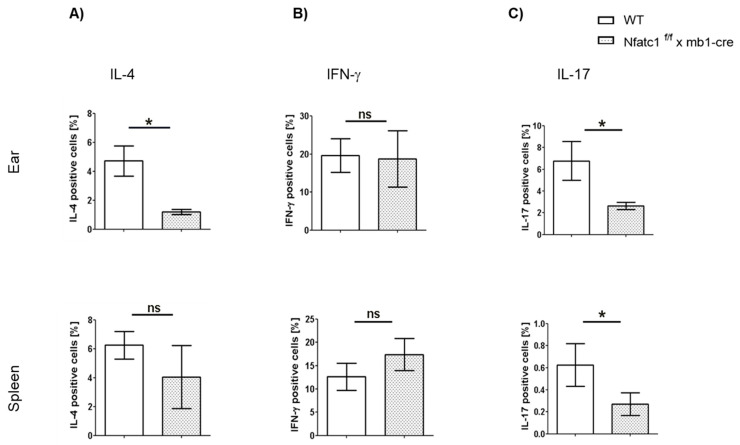
Differential cytokine production by CD4+ T cells in the ear skin (**A**–**C**) or spleen (**A**–**C**) of *Nfatc1^f/f^ x mb1-cre* mice versus WT animals during the CHS response. Significantly reduced IL-4- and IL-17-producing CD4^+^ cells along with unaltered IFN-γ production were observed at the site of inflammation (ear) in *Nfatc1^f/f^ x mb1-cre* mice compared with WT mice. * *p* < 0.05, ns, not significant *p* > 0.05.

We observed that CHS to TNCB was significantly diminished in *Nfatc1^f/f^ x mb1-cre* mice compared with WT mice (Figure 1 and Figure 2). These results are supported by the increased numbers of Bregs along with pronounced IL-10 production in NFATc1-ablated B cells, thereby potentially suppressing IL-4- and IL-17-producing CD4^+^ T cells (Figure 3, Figure 4 and Figure 5). In ACD and other related skin inflammatory disorders, IL-4 and IL-17 are the key cytokines responsible for their pathophysiology [28,29,30]. A subset of B cells (CD5^+^CD1d^hi^) has been described as Bregs, which are considered to tune immune reactions via IL-10 secretion and exert a regulatory influence on different T cell subsets [16,31,32,33]. Published data from CD1d knockout mice indicated robust CHS responses to the reduced IL-10 production by Bregs [34]. However, it is unclear whether Bregs correspond to a specific B cell subset that can be converted to IL-10-producing B cells during inflammatory signals [35]. Regulatory B cells have been shown to suppress the effector CD4^+^ T cell responses—including Th2 and Th17 differentiation—by both cytokine-dependent and contact-dependent mechanisms [15,32,36]. Interestingly, Bhattacharya et al. have demonstrated that NFATc1-deficient B cells exhibit a reduced capacity to stimulate T cell proliferation and IL-2 synthesis, along with impaired antigen presentation. In vivo, these Bregs lead to a milder clinical course of experimental autoimmune encephalomyelitis (EAE), a T cell-mediated disease [15]. Bregs maintain the balance between Tregs and Th1/Th17 populations; in addition, they prevent the differentiation of naive T cells into Th1 and Th17 while inducing Treg differentiation and cell expansion, which further suppresses pathological Th1 and Th17 responses [37]. In line with this, we have recently shown that mice deficient in NFATc1 in T cells have more regulatory T cells during CHS responses. NFATc1-deficient CD8^+^ T cells have a reduced potential of polarization into IL-17A-producing Tc17 cells. By in vivo adoptive cell transfer, we confirmed that NFATc1-deficient CD8^+^ T cells were unable to induce a CHS response in recipient mice upon TNCB exposure. Using transcriptomic analyses, we reported a reduced expression of the RoR-γ gene in NFATc1-deficient CD8^+^ T cells along with a reduced production of IL-17A, indicating the importance of NFATc1 for Tc17 differentiation. Moreover, single-cell RNA sequencing of WT and NFATc1-deficient in vitro polarized Tc17 cells revealed that NFATc1 favors the phenotypic differentiation of CD8^+^ T cells into Tc17 cells. Additionally, our data showed that NFATc1 ablation is likely to enhance the differentiation of IFN-γ-producing CD8+ (Tc1) cells. However, we observed no significant impact of such increase in Tc1 on induction of CHS responses [23]. Therefore, we believe that IL-17-producing T cells are major players in inducing early CHS responses while any perturbance in their function, e.g., by increased IL-10-producing Bregs, alleviates the allergic response. Accordingly, we observed that IL-17 was significantly decreased in both the ear skin and spleen during CHS responses in *Nfatc1^f/f^ x mb1-cre* mice, confirming their important role in inflammatory responses (Figure 5).

While our study focused on the resolution phase (day 8 post challenge), future experiments assessing the temporal dynamics of IL-10^+^ B cells across the sensitization and elicitation phases should help to provide a more comprehensive view of their role in CHS regulation. Longitudinal sampling could help distinguish between early-induction and late-stage regulatory effects. Such data of the inhibition of IL-10 expression via NFATc1 might have a general impact on the development of allergies. The interplay between NFATc1 activity and IL-10 expression is not restricted to allergic responses but also appears to be relevant in other autoimmune and inflammatory diseases [16,38].

In conclusion, our findings have shown that NFATc1 serves as a molecular switch that interferes with the CHS response by mediating the polarization of B cells into IL-10-producing Bregs. The latter cells appear to control the balance between T cell subsets during allergic inflammatory responses by decreasing the number of IL-17^+^ CD4^+^ T cells. Our data support the notion that targeting NFATc1 during allergic reactions may be explored for future therapeutic strategies in allergic diseases.

## 4. Material and Methods

### 4.1. Mice, Isolation, and Culture of Cells

We used 8- to 12-week-old C57BL/6 mice in this study. *Nfatc1^f/f^ x mb1-cre* mice have been described previously [39]. Briefly, to generate mice with *Nfatc1*-depleted B cells, *Nfatc1^f/f^* mice were crossed with mice (C57/BL6) that specifically expressed the cre enzyme in bone marrow B cells [19]. Wild-type C57BL/6 animals served as controls. The mice were bred and maintained in the Central Animal Facility of the Medical Faculty (ZEMM), University of Würzburg, according to legal regulations. Animal experimental procedures and protocols were approved by the responsible ethical committee of Regierung von Unterfranken, Würzburg (license number 55.2.2-2532.2-789). Mice were assigned to groups based on their genotype. All experiments were conducted at least three times with each group consisting of 5 animals (*n*  =  15). Sensitization and elicitation was performed using 2,4,6-trinitrochlorobenzene (TNCB) according to the standard CHS model [40]. Mice were shaved at the abdomen (area of 2 × 2 cm) with an electric shaver (Braun, Kronberg, Germany). Three % TNCB in acetone was applied at day 0 to the abdomen followed by the application of 1% TNCB on both ears at day 5. Ear swelling was measured using a caliper (Käfer, Villingen-Schwenningen, Germany) on days 5–8. For the controls, the mice were left non-sensitized followed by elicitation by applying TNCB at day 5. The IL-10^+^ Breg and other cellular analyses were performed on day 8 to capture the regulatory phase of the CHS response. Previous studies have shown that IL-10^+^ regulatory B cells accumulate after the peak of inflammation to promote immune resolution and tissue homeostasis [20,41].

After euthanasia, the spleen, ears, and draining lymph nodes were collected and partly fixed with paraformaldehyde or used for cell isolation. Ears were cut into small pieces and washed with PBS before incubation with 5 mL Accumax (Sigma, Saint Louis, MO, USA) for 30 min. The samples were transferred to c-tubes (Miltenyi Biotec, Bergisch Gladbach, Germany) and serum-free medium was added. Tubes were inserted into the Gentle MACS dissociator (Miltenyi Biotec) and incubated for 36 s. The cell solution was poured over a 70 µm cell strainer into 50 mL Falcon tubes, centrifuged, and washed with PBS. B cell isolation was performed using a B cell isolation kit according to the manufacturer’s instructions (Miltenyi Biotec). Splenocytes and B lymphocytes were cultured in 6- or 24-well-plates and stimulated with either Brefeldin A (20 µg/mL), Ionomycin (1 µg/mL) and PMA (phorbol 12-myristate 13-acetate, 100 ng/mL) (Sigma, Saint Louis, MO, USA) for 6 h or incubated with CpG-ODN (1 µg/mL) (InvivoGen, San Diego, CA, USA) and CD40 ligand (1 µg/mL) (Invitrogen, Waltham, MA, USA) for 48 h followed by 6 h of incubation with Brefeldin A, PMA and Ionomycin. After incubation, cells were processed for flow cytometry.

### 4.2. Flow Cytometry

Lymphocyte staining for flow cytometry (CytoFlex LX, Beckmann Coulter, Brea CA, USA) was performed using mAbs recognizing the following targets: CD3 FITC (Biolegend, San Diego, CA, USA, #100204), CD4 PerCP (Biolegend, #121416), IL17A BV421 (Biolegend, # 506925), IL4 PE (Biolegend, #504104), IL10 PE-Dazzle 594 (Biolegend, #505034), IFNy PE-Cy7 (Biolegend, #505826), CD8 BUV395 (BD, #565968). CD45R/B220 AF700 (Biolegend, #103323), CD19 BV785 (Biolegend, #115543), CD1d PE (Biolegend, #123510), CD5 APC (Biolegend, #100626). Data were analyzed by FlowJo™ v10.8 Software (BD Life Sciences, Franklin Lakes, NJ, USA). The gating strategy for flow cytometry analysis is shown in Supplementary Figure S1.

### 4.3. Histological and Immunofluorescence Staining

Skin specimens from wild-type and *Nfatc1^f/f^ x mb1-cre* mice were fixed in 4% paraformaldehyde and embedded in paraffin. Microtome-cut 3 µm tissue sections were treated with xylene and absolute ethanol before staining in hematoxylin solution and counter-staining with eosin according to manufacturer’s instructions.

### 4.4. Statistical Analysis

The statistics evaluation was performed using GraphPad (Prism) software, version 6.0. Data are presented as mean ± SEM. An unpaired *t*-test was applied to calculate statistical significances; these are indicated as *** *p* < 0.001, ** *p*< 0.01, and * *p* < 0.05.

## Data Availability

All data are contained in this article and there are no repository data.

## Supplementary Materials

The following supporting information can be downloaded at: https://www.mdpi.com/article/10.3390/ijms26178125/s1.
