# Supplementary material for: NFATc1 Abrogation in B Cells Ameliorates Contact Hypersensitivity Responses
Source: Int J Mol Sci. 2025 Aug 22;26(17):8125. doi: 10.3390/ijms26178125 (PMC12428475; doi:10.3390/ijms26178125)
Supplement: Supplementary file 1 [file ijms-26-08125-s001.zip › ijms-3763352-supplementary.pdf]

A)

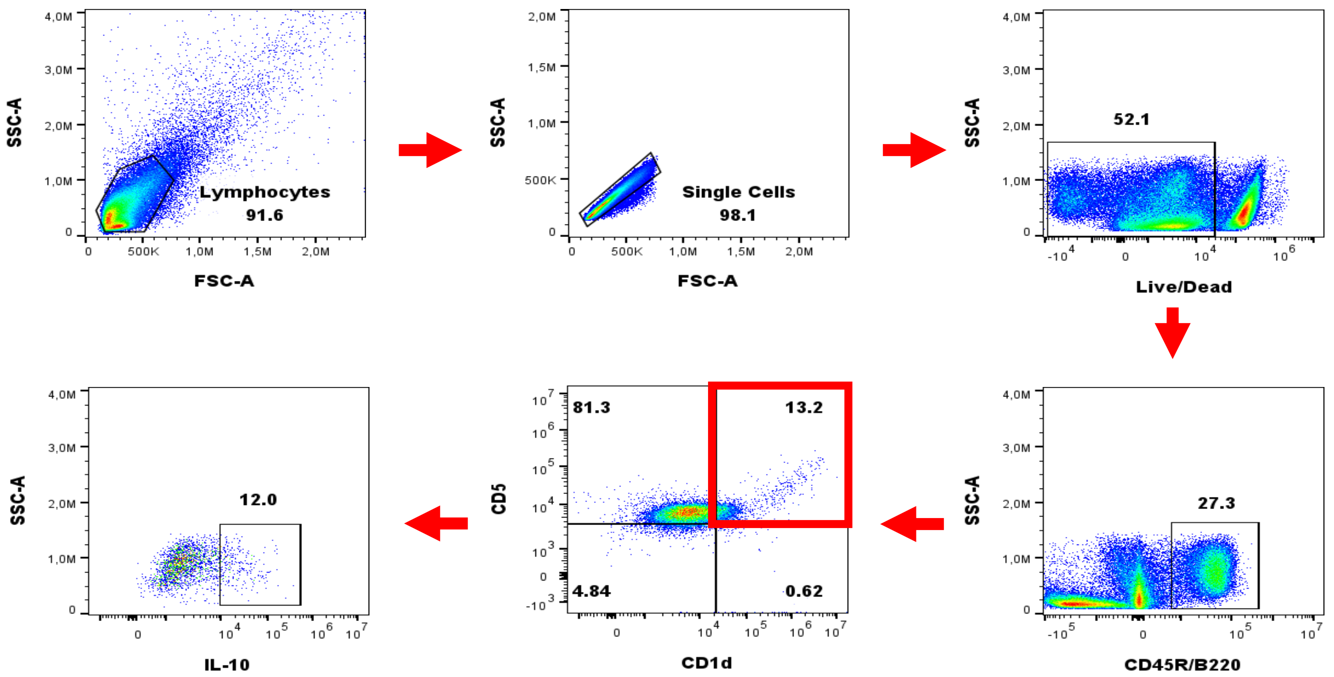

B)

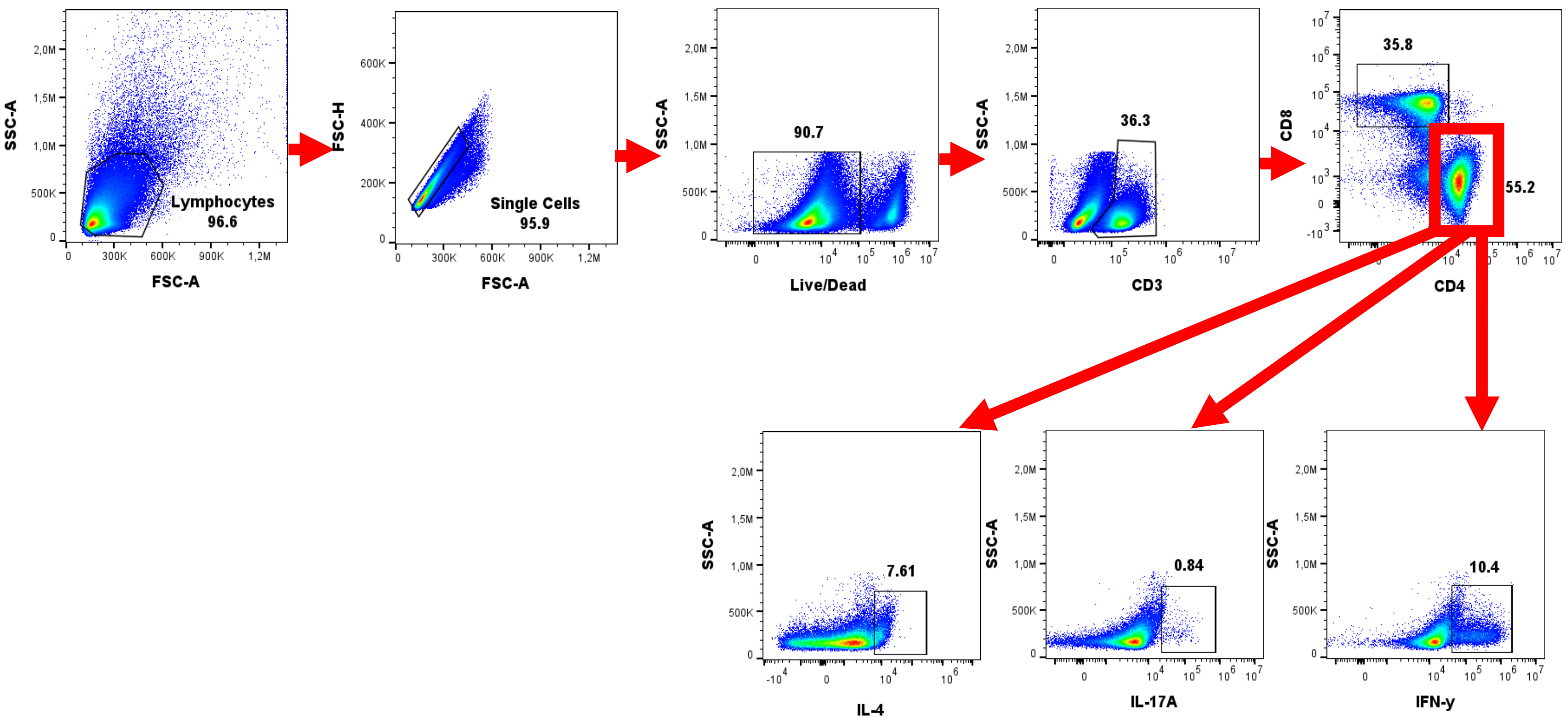

Supplementary Figure S1: Flow cytometric gating strategy. **A)** Regulatory B cells expressing CD5, CD1d and IL-10. **B)** IL-4-, IL-17- and IFN-γ-producing CD4+ T cells.
